# Down-regulation of protease-activated receptor 2 ameliorated osteoarthritis in rats through regulation of MAPK/NF-κB signaling pathway *in vivo* and *in vitro*

**DOI:** 10.1042/BSR20192620

**Published:** 2020-03-25

**Authors:** Shichang Yan, Huimin Ding, Junyang Peng, Xinqiang Wang, Chenglong Pang, Juncheng Wei, Jianjun Wei, Hui Chen

**Affiliations:** Department of Orthopaedic, BenQ Medical Center, The Affilicated BenQ Hospital of Nanjing Medical University, 210019, Nanjing, Jiangsu Province, China

**Keywords:** Autophagy, MAPK/NF-kB signaling pathway, Osteoarthritis, Protease-activated receptor 2

## Abstract

Recently, protease-activated receptor 2 (PAR2) has been proved to be involved in the inflammatory response including osteoarthritis (OA). In the present study, we found that PAR2 antagonist could remarkably improve the pathological condition of OA rats *in vivo*. In addition, we also found that PAR2 antagonist could suppress the production of inflammatory factors (TNF-α and Cox-2), decrease the levels of MMP-1 and MMP-13, and restrain the levels of P62 proteins and aggravate the expression of LC3-II both *in vivo* and *in vitro*. Besides, *in vitro*, PAR2 antagonist could increase the proliferation and colony formation of chondrocytes induced with IL-1β. Moreover, PAR2 antagonist could decrease the expression of expressions of p-p38, p-IκBα and p-NF-κB *in vitro*. However, PAR2 agonist exhibited the opposite effects. Furthermore, SB203580, a p38 MAPK inhibitor, could remarkably promote the proliferation of chondrocytes induced with IL-1β, could alleviate the production of TNF-α and Cox-2, could down-regulate the protein expressions of MMP-1 and MMP-13, and could decrease the expression of P62 and increase the expressions of LC3-II of chondrocytes induced with IL-1β. Importantly, SB203580 could reverse the effects of PAR2 agonist on the functions of chondrocytes induced with IL-1β. Taken together, the present data suggest that down-regulation of PAR2 can ameliorate OA through inducing autophagy via regulation of MAPK/NF-κB signaling pathway *in vivo* and *in vitro*, and PAR2 can be considered as a potential candidate to treat OA.

## Introduction

Osteoarthritis (OA) is a common inflammatory joint disease in orthopaedics [1,2]. With the aging and obesity of population, the incidence rate of OA is increasing year by year, and OA causes heavy burdens for individuals, families and society [3]. OA often occurs in weightbearing joints and joints with frequent movement, such as hip joint, knee joint, ankle joint, temporomandibular joint and so on, and the lesion area mainly includes synovial tissues, cartilage tissues, subchondral bone, the soft tissues around the shoulder muscles and ligaments, meniscus and infrapatellar fat pad [4]. The main pathological features are decreased number of chondrocytes in joint tissues, metabolic disorder of extracellular matrix, inflammatory response of synovial membrane, remodeling of subchondral bone and so on [5], and these pathological changes will lead to joint deformation and joint dysfunction in patients with OA, resulting in a mortality rate of up to 53% [6]. Clinical treatment of OA is to relieve joint pain, maintain or improve the physiological function of the joint, and protect the structure of the joint [7]. At present, there are many methods to treat OA with different effects, but OA is mainly treated by stages according to the pathological progress, including conservative medical treatment in the early stage of OA and arthroplasty in the late stage of OA. Early clinical medication is often limited to symptoms, and cannot effectively prevent the occurrence and development of OA diseases, even usually accompanied by gastrointestinal, cardiac and other side effects [8,9]. In the late stage of OA, arthroplasty is almost the only alternative treatment, while it is risky and costly. However, theoretically promising tissue engineering research is still far from clinical application [10,11]. Therefore, it is a long-term, severe and urgent task to find safe, reliable and effective treatment for OA.

Recent studies have found that protease-activated receptor 2 (PAR2) was involved in the occurrence and development of OA by influencing the inflammatory response of cartilage and synovium, and the balance of cartilage matrix decomposition and anabolism [12,13]. Importantly, PAR2 plays a key role and is the only member of the PAR family that has been proved to be involved in the inflammatory response including OA [14–16]. For example, PAR2 activation could induce inflammatory changes such as joint swelling and vasodilation in wild-type mice, while almost no similar manifestations were found in PAR2 gene knockout model of arthritis in mice, suggesting that abnormal activation of PAR2 can cause joint lesions and contribute to the occurrence and development of OA [17]. Besides, Huesa et al. found that PAR2 transfection could aggravate cartilage damage and induce osteophyte formation in wild-type and PAR2-deleted mice, indicating that OA-related changes in bone and cartilage depended on the role of PAR2 and were regulated by PAR2 [18]. Further, Milner et al. have shown that PAR2 was the target of proteolytic enzymes in OA cartilage, and inhibiting PAR-2 expression can effectively block collagen lysis induced by proteolytic enzymes and alleviate cartilage damage [19]. Therefore, PAR2 plays an important role in cartilage homeostasis during OA progress.

At present, autophagy has been found to be involved in many biological processes, including cell survival, aging and death. Autophagy plays a very wide role in physiology and pathophysiology, involving metabolic diseases, tumors, degenerative diseases, aging, infection, immunity and so on [20,21]. Autophagy may be involved in cartilage aging and degeneration in osteoarthritis [22]. Carames et al. found that autophagy may be a protective mechanism in normal cartilage to avoid cell death caused by aging and trauma [23]. By inducing mouse osteoarthritis model, the research team detected the decreased expression of unc-51 like autophagy activating kinase 1 (ULK1), Beclin1 and microtubule Associated Protein 1 Light Chain 3 (LC3), and proposed that the autophagic compensatory mechanism of chondrocyte was a new pathogenesis of OA. Autophagy is a dynamic balance mechanism for removing dysfunctional organelles and macromolecules, and its enhancement may be a new way to delay joint aging and reduce risk factors of osteoarthritis [24]. Sasaki et al. proposed that chondrocyte autophagy can regulate the production of reactive oxygen species (ROS) and the expression of OA-related genes such as Aggrecan, Collagen Type II Alpha 1 (Col2A1), matrix metallopeptidase 13 (MMP13) and ADAM metallopeptidase with thrombospondin type 1 motif 5 (ADAMTS5) [25]. In addition, Almonte-Becerril et al. performed immunohistochemical and Western blot assays to determine cell apoptosis (active caspase 3 and TUNEL signals) and autophagy (LC3II molecules and cytoplasmic vacuoles) in animal OA models, confirming the co-existence of autophagy and apoptosis in chondrocyte death [26]. The latest findings have shown that PAR2 could induce kidney tubular epithelial inflammation by inhibiting autophagy via the PI3K/Akt/mTOR signaling pathway [27].

Based on these, we speculated that PAR2 and autophagy may play essential effects on the pathogenesis in OA. Therefore, the article was designed to explore the role of PAR2 and chondrocyte autophagy in OA, and further elucidated the underlying mechanisms of PAR2 in regulating chondrocyte autophagy, providing a theoretical basis for PAR2 to become a therapeutic point in OA.

## Materials and methods

### Experimental animals

A total of 24 Sprague-Dawley (SD) rats (weighing 200 ± 20 g) were purchased from Shanghai Laboratory Animal Research Center, including 12 male rats and 12 female rats. All the experimental rats were kept under specific pathogen-free conditions with free access to food and water. All experiments were performed in Nanjing Medical University, and followed the Guidelines. Animals were cared for in accordance with Guide for the Care and Use of Nanjing Medical University.

### Establishment of OA models in rat and animal grouping

The rat models of OA were established with transection of anterior cruciate ligament (ACL) as previously published [28]. The rats were anesthetized with 2% pentobarbital sodium solution via intraperitoneal injection (40 mg/kg). The rats were fixed on the operation table and an incision (1 cm) was made in the medial side of the right knee joint to expose the medial collateral ligament, which was then cut off. Subsequently, the joint capsule was opened and primary lesion in the joint cavity was examined. Further, the ACL was transected and the medial meniscus was completely cut off. Moreover, the incisions were sutured layer by layer, bandaged under sterile conditions and fixed. Following the operation, antibiotic was administered the next day to prevent infection. One week after the operation, rats in the OA group and the normal group were conventionally kept. All the animals were killed with an overdose injection of pentobarbital sodium (200 mg/kg).

To observe the role of PAR2 in cartilage of OA rats, all the experimental rats were divided into four groups (*n* = 6), including normal group (normal rats without any treatment), OA group (OA rats without any treatment), PAR2 agonist group (OA rats injected with 100 µl SLIGRL-NH_2_ (10 µg/100 µl) through knee joint cavity) and PAR2 antagonist group (OA rats injected with 100 µl FSLLRY-NH_2_ (10 µg/100 µl) through knee joint cavity).

### Hematoxylin–eosin (HE) staining

The cartilage tissues were taken from each rat in each group, fixed in 10% formalin, and then embedded in paraffin, and sequential serial sections were obtained. Afterward, the sections were stained with hematoxylin–eosin (HE) staining. Images were obtained using a light microscope (Leica Microsystems, Wetzlar, Germany).

### Immunohistochemical analysis

The isolated cartilage tissues were fixed in 4% neutral formalin for 24 h, embedded in paraffin and serially sectioned at 5 µm. Further, the sections were deparaffinized and rehydrated, then submerged in hydrogen peroxide to quench peroxidase activity following incubated with 1% BSA to block non-specific binding sites. Afterward, the primary antibodies were incubated at 4°C for 12 h, and then secondary antibodies were applied for another 1 h at room temperature. All the sections were visualized using diaminobenzidine (DAB, Beyotime) under a light microscope (Leica Microsystems, Wetzlar, Germany). Images were taken at 200× magnification and the scale bar = 50 μm. Antibodies in immunohistochemical analysis were purchased Cell Signaling Technology (Beverly, MA, U.S.A.), including LC3-II (#3868) and P62 (#23214).

### Cell isolation and culture

Chondrocytes were isolated from the knee joints of 7-day-old SD rats. In brief, rats were killed by an overdose of pentobarbital and the articular cartilages were collected from the knee joints. Then, cartilages were cut into pieces and digested with 2 mg/ml of collagenase II for 3 h at 37°C. Finally, the collected chondrocytes were suspended in complete Dulbecco’s Modified Eagle’s Medium (DMEM) supplemented with 10% fetal bovine serum (FBS, Gibco, U.S.A.), 100 mg/ml streptomycin (Gibco, U.S.A.) and 100 U/ml penicillin (Gibco, U.S.A.), and cultured in 5% CO_2_ atmosphere at 37°C.

### Cell treatment

Chondrocytes were cultured in 96- or 12-well plates at 80% density and induced with IL-1β (10 ng/ml) as a cell inflammatory model, and the normal chondrocytes (NC) were used as control. About 20 µM rapamycin, 5 mM 3-Methyladenine (3-MA), PAR2 agonist (50 µM SLIGRL-NH_2_) or PAR2 antagonist (50 µM FSLLRY-NH_2_) was used to stimulate the cell inflammatory model, and then related detections were performed. Further, PAR2 agonist (50 µM SLIGRL-NH_2_) or/and p38 MAPK inhibitor (10µM SB203580) were used to stimulate the cell inflammatory model, and then related detections were performed.

### Cell counting kit-8 (CCK-8) assay

The viabilities of chondrocytes were determined by CCK-8 assay. Briefly, chondrocytes were seeded in 96-well plates at a density of 1 × 10^4^ cells/well, and incubated with different treatments in a humidified incubator at 37°C for 0, 24 and 48 h, respectively. Then, 10 μl of CCK-8 (Sigma Chemical Co, St Louis, MO, U.S.A.) was added to each well for another 2 h at 37°C. The optical density (OD) was recorded at 450 nm using a microplate reader (Dojindo Molecular Technology, Rockville, MD, U.S.A.).

### EdU assay

5-ethynyl-2′-deoxyuridine (EdU) incorporation proliferation assay was carried out to evaluate the proliferation of chondrocytes using a Cell-Light™ EdU Imaging detecting kit (RiboBio, Guangzhou, China). Chondrocytes were seeded in 6-well plates and incubated for 24 h after different treatments. All of the EdU incorporation experiments were performed according to the manufacturer’s protocol. The ratio of EdU-positive nuclei to total nuclei was calculated as the proliferation rate of cells in six random high-power fields per well. The cells were visualized by a fluorescence microscopy (Olympus, Tokyo, Japan).

### Colony formation analysis

Colony formation assay was conducted to evaluate the role of PAR2 in the proliferative potential of chondrocytes. Chondrocytes at a density of 1 × 10^3^ cells/well were plated in 6-well plates, cultured at 37°C with 5% CO_2_, and the medium was replaced every 2–3 days. After 2 weeks, the plates were fixed with 4% paraformaldehyde for 20 min and stained using 10% Crystal Violet for 30 min. Then, the number of stained colonies was manually counted.

### Enzyme-linked immunosorbent (ELISA) assay

The concentrations of cytokines in isolated cartilage tissues and chondrocytes were examined by ELISA for rat IL-1β, TNF-α and COX2 (eBioscience, San Diego, CA) following the manufacturer’s instructions.

### Western blotting assay

The total protein of isolated cartilage tissues and chondrocytes was extracted according to the manufacturer’s recommended protocol (Vazyme, U.S.A.), and the protein concentrations were determined using the BCA Protein Assay Kit (Vazyme, U.S.A.). Samples with equal amounts of protein (50 μg) were fractionated on 10% SDS polyacrylamide gels, transferred to polyvinylidene difluoride membranes (PVDF), and blocked in 5% skim milk in TBST for 1.5 h at 25 ± 1°C. The membranes were then incubated at 4°C overnight with 1: 1000 dilutions (v/v) of the primary antibodies. After washing the membranes with TBST, incubations with 1:1000 dilutions (v/v) of the secondary antibodies were conducted for 2 h at 25 ± 1°C. Protein expression was detected using an Enhanced Chemiluminescence Detection System. GAPDH was used as a loading control. Antibodies in Western blot were purchased Cell Signaling Technology (Beverly, MA, U.S.A.), including TIMP1 (#8946), MMP-13 (#94808), LC3-I (#4599), LC3-II (#3868), P62 (#23214), PAR2 (#6976), p38 MAPK (#8690), phosphorylation (p)-p38 (#4511), IκBα (#4814), p-IκBα (#2859), NF-κB (#8242), p-NF-κB (#3033), Lamin B1 (#13435), Cox-2 (#12282), GAPDH (#5174).

### Statistical analysis

GraphPad Prism 5 software was used to carry out all statistical analyses. Data were shown as mean ± standard deviation. Student’s *t*-test was used to compare the difference between two groups, one-way ANOVA analysis followed by Tukey’s poc host test was used to compared the difference between multiple groups. *P* values < 0.05 were considered statistically significant. All experiment was carried out at least three times.

## Results

### Down-regulation of PAR2 ameliorated OA in rats

**Figure 1:**
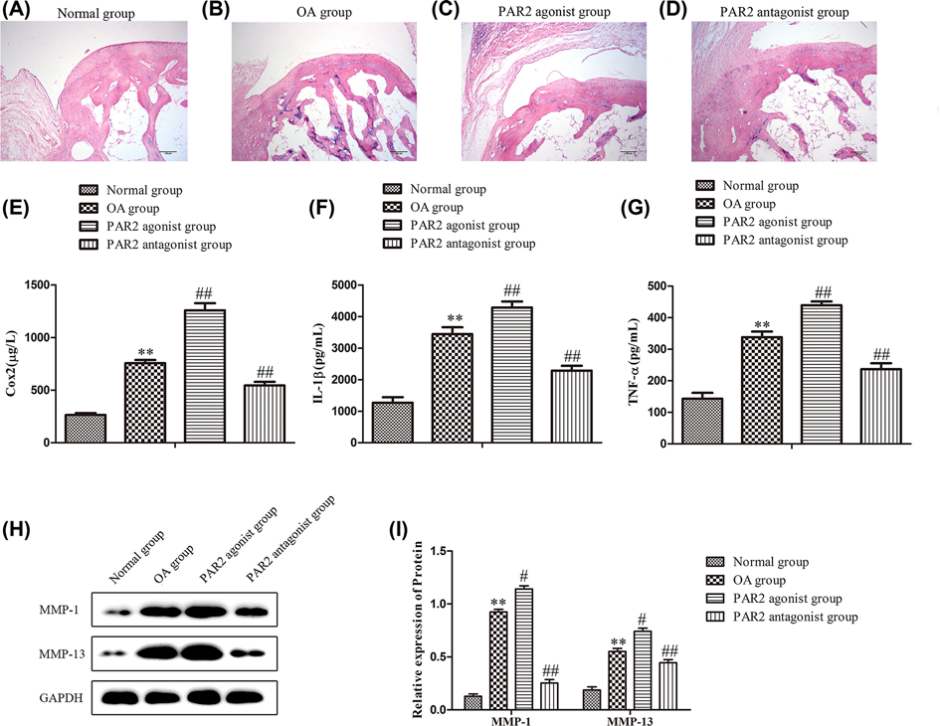
Pathology, inflammatory factors, matalloproteinases were regulated by PAR2 in osteoarthritis cartilage tissue in rats OA rats were injected with PAR2 agonist or PAR2 antagonist through knee joint cavity (**A**–**D**) The pathological changes were detected by HE staining assay. (**E**–**G**) The levels of Cox-2, IL-1β and TNF-α were determined using ELISA. (**H,I**) The levels of MMP-1 and MMP-13 were determined using Western blot and quantification analysis. The values are mean ± SD of three independent experiments. **P<0.01 vs normal group, ^##^*P<*0.01 vs OA group.

In order to evaluate the role of PAR2 in the development and progress of OA, we established OA models in rats which were administrated with PAR2 agonist and PAR2 antagonist, respectively. First, HE staining assay was performed to examine the pathological changes of OA in rats of each group. As shown in Figure 1A–D, the articular cartilage from the normal group was stained pink, articular space was clearly visible, and articular surface was smooth. While in OA group, the articular surface was uneven, pink articular cartilage almost disappeared, the cellular structure was disordered, the articular space was very narrow, and more fibroblasts fillings were observed. After treatment with PAR2 antagonist, the articular space was slightly narrow, but still clearly visible, pink articular cartilage was clearly visible, but partially destroyed. The pathological changes of articular cartilage from the PAR2 agonist group were more serious.

In addition, ELISA assay was carried out to determine the effects of PAR2 on the secretion of inflammatory factors. As indicated in Figure 1E,F,G, compared with normal group, the expressions of IL-1β, TNF-α and Cox-2 in OA group were significantly up-regulated, while treatment with PAR2 agonist could promote the section of IL-1β, TNF-α and Cox-2, and administration with PAR2 antagonist remarkably suppressed the levels of IL-1β, TNF-α and Cox-2 related to those in OA group.

Further, abnormal expressions of matrix metalloproteinase (MMPs) were the cause of ECM degradation and the important cause of degenerative cartilage lesions in OA [29]. Therefore, Western blot assay was adapted to detect the expressions of MMP-1 and MMP-13. The results showed that the expressions of MMP-1 and MMP-13 were significantly over-expressed in OA group when compared with those in the normal group, while the levels of MMP-1 and MMP-13 were enhanced in PAR2 agonist group, and the levels of MMP-1 and MMP-13 were decreased in PAR2 antagonist group related to those in the OA group (Figure 1H,I). These data suggested that PAR2 may play an important role in the development and progress of OA, and down-regulation of PAR2 ameliorated OA.

### Down-regulation of PAR2 promoted autophagy in cartilage tissues in OA rats

**Figure 2:**
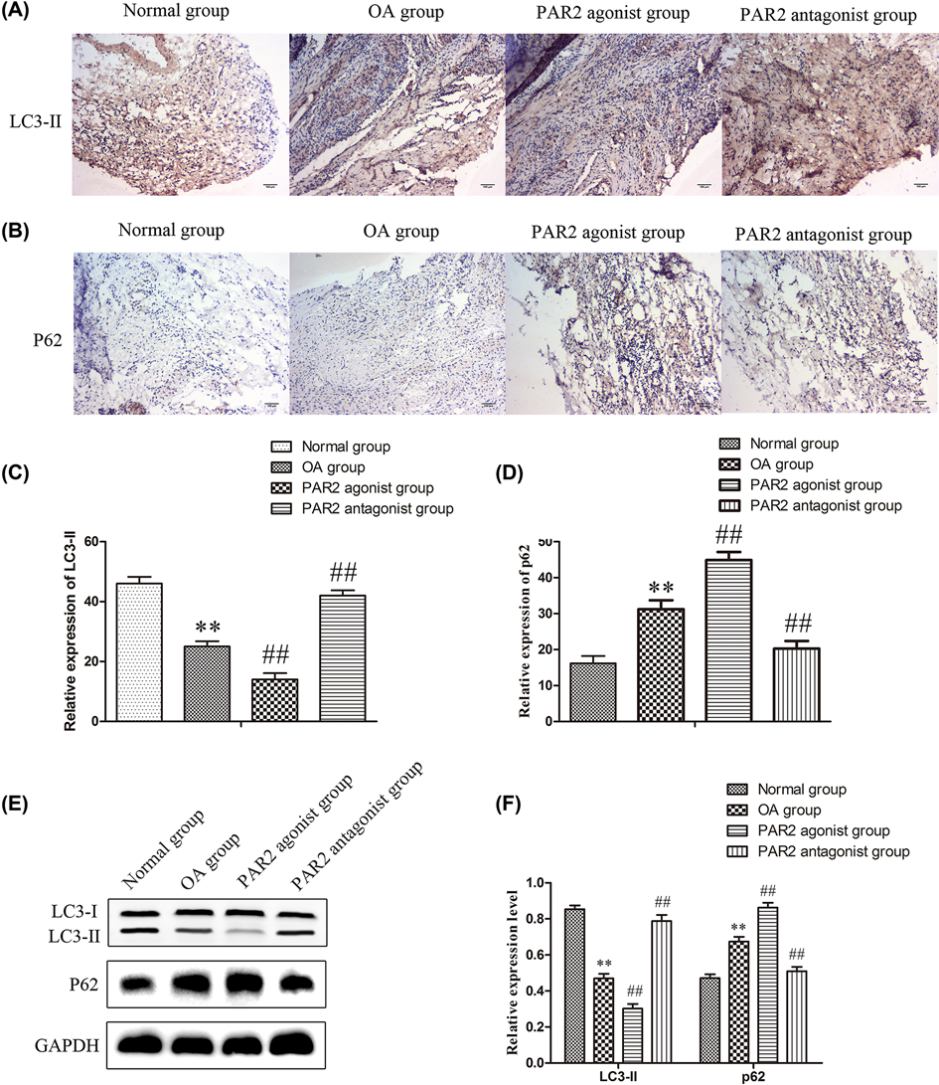
Relative protein of autophagy was regulated by PAR2 in osteoarthritis cartilage tissue in rats (**A** and **C**) The expression of LC3II was detected by immunohistochemistry and quantification analysis. (**B** and **D**) The expression of p62 was detected by immunohistochemistry and quantification analysis. (**E** and **F**) The expressions of LC3II and p62 were examined by Western blot and quantification analysis. The values are mean ± SD of three independent experiments. **P<0.01 vs normal group, ^##^*P*<0.01 vs OA group.

Recently, autophagy has been found to be involved in many biological processes in a large number of organisms, and autophagy may be involved in the aging and degeneration of articular cartilage in OA [30,31]. To explore whether PAR2 was involved in autophagy of articular cartilage, immunohistochemistry were performed to assess the expressions of LC3-II and P62 related to autophagy. As shown in Figure 2A–D, the protein expression of P62 was up-regulated and the protein expression of LC3-II was significantly down-regulated in OA group when compared with those in normal group. The expression levels of P62 protein were enhanced and the expression levels of LC3-II was significantly inhibited in PAR2 agonist group, while the expression levels of P62 protein were significantly suppressed and the expression levels of was significantly promoted in PAR2 antagonist group when compared with those in OA group. The results of Western blot were consistent with that of immunohistochemistry (Figure 2E,F). These data indicated that PAR2 exhibited the effects on OA which may be achieved by regulating autophagy of articular cartilage.

### Down-regulation of PAR2 promoted proliferation in chondrocytes induced with IL-1β

**Figure 3:**
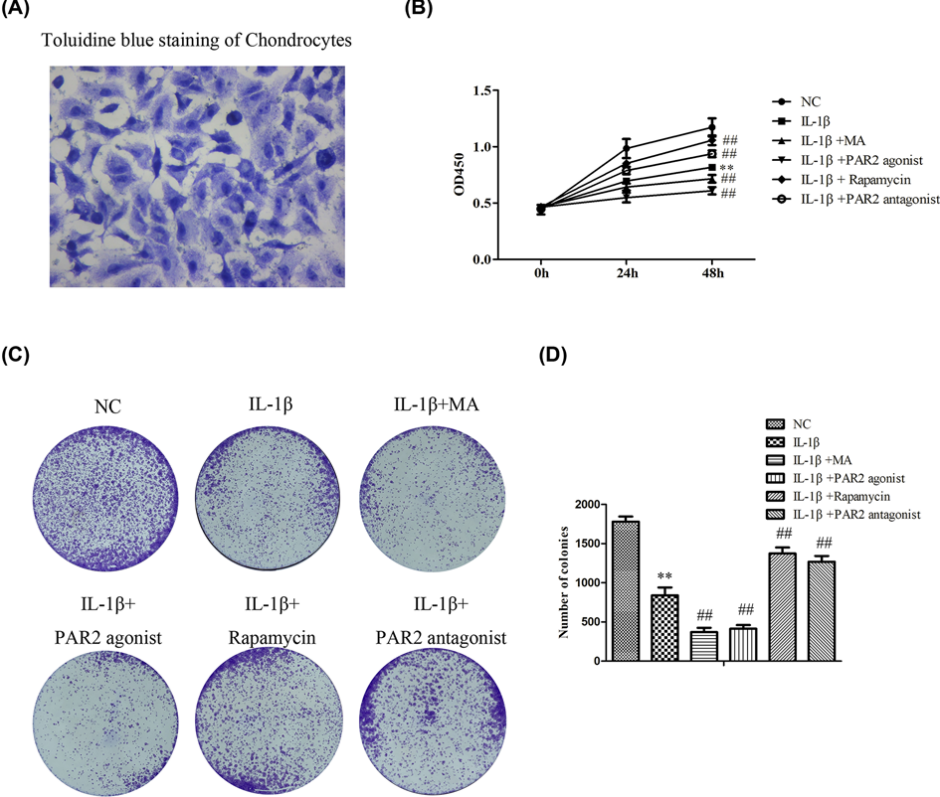
Cell proliferation is regulated by PAR2 in chondrocytes induced by IL-1β (**A**) Primary chondrocytes of rats were extracted, and toluidine blue staining were performed to identify primary chondrocytes. (**B**) The cell viability was determined by CCK-8 assay. (**C** and** D**) The cell proliferation was determined by clony formation assay. The values are mean ± SD of three independent experiments. **P<0.01 vs NC group, ^##^*P*<0.01 vs IL-1β treatment group.

To further explore the effects and underlying mechanisms of PAR2 in the development and progress of OA, we extracted the primary chondrocytes of rats. As shown in Figure 3A, toluidine blue staining were performed to identify primary chondrocytes, and the results showed that after toluidine blue staining, acidic mucous substances in chondrocytes and extracellular matrix were dyed blue-purple, nucleic acid and other substances were dyed dark blue. Microscopically, blue heterochromatic granules were observed in the cells. To sum up, the successful culture of chondrocytes was identified.

In order to study the effects of PAR2 on the proliferation of chondrocytes induced with IL-1β, first, CCK-8 assay was performed. As shown in Figure 3B, IL-1β could significantly suppress the proliferation of chondrocytes, PAR2 antagonist and Rapamycin could promote the proliferation of chondrocytes induced with IL-1β, while PAR2 agonist and 3-methyladenin (3-MA) could notably suppress the proliferation of chondrocytes induced-with IL-1β. Moreover, to evaluate whether PAR2 regulated the colony formation of chondrocytes, cells were treated with PAR2 antagonist, PAR2 agonist, Rapamycin and 3-MA and incubated for 2 weeks. The results of Figure 3C,D showed that compared with normal group, IL-1β could significantly suppress colony formation of chondrocytes. However, PAR2 antagonist and Rapamycin could increase colony formation of chondrocytes induced with IL-1b, and PAR2 agonist and 3-MA remarkably decreased colony formation of chondrocytes induced with IL-1β related to those in IL-1β group. These data indicated that PAR2 could affect the proliferation of chondrocytes induced with IL-1β which may be achieved by regulating autophagy of chondrocytes.

### Down-regulation of PAR2 suppressed the production of inflammatory cytokines and the expressions of MMPs, and induced autophagy in chondrocytes induced with IL-1β

**Figure 4:**
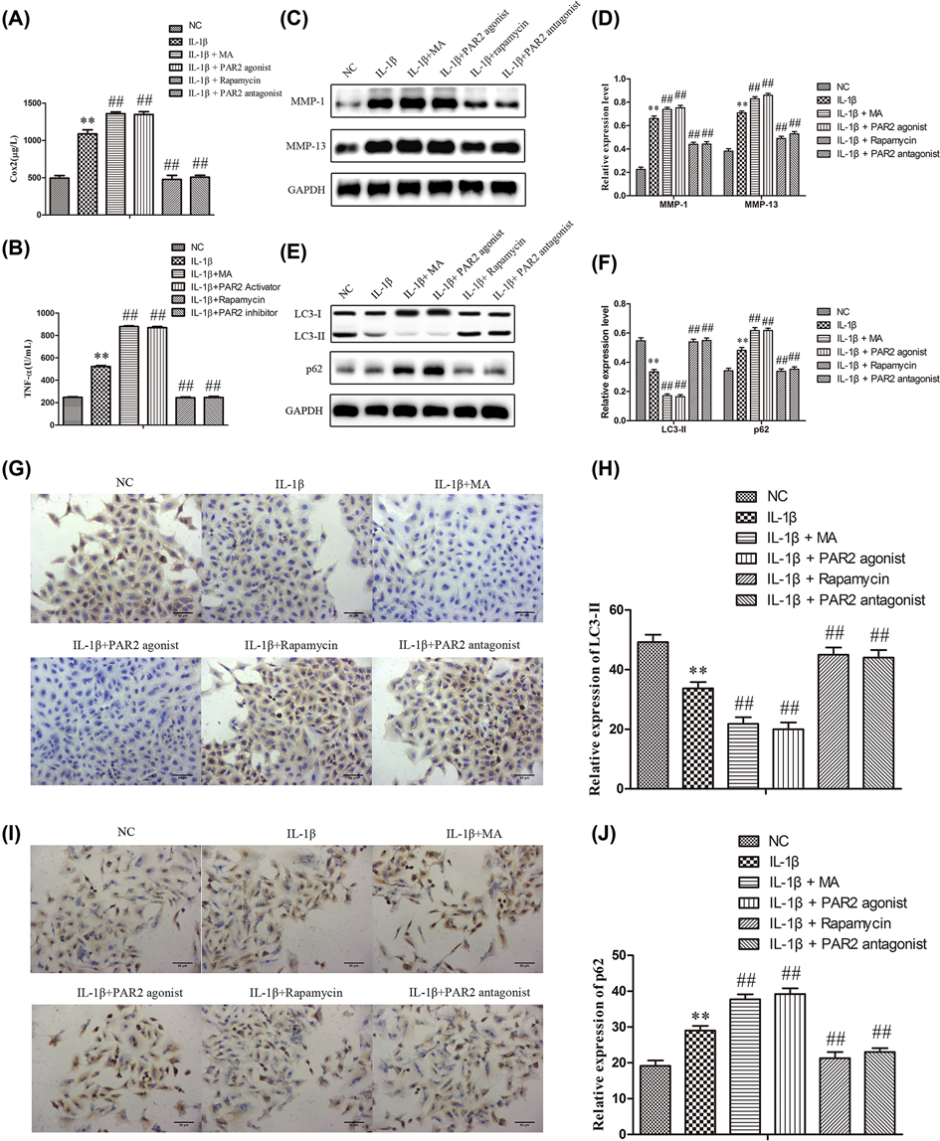
Inflammation factor, matrix protease and relative protein of autophagy were regulated by PAR2 in chondrocytes induced by IL-1β (**A** and** B**) Inflammation factor (Cox-2 and TNF-α) were determined using ELISA. (**C** and **D**) matrix protease (MMP-1 and MMP-13) were detected by Western blot and quantification analysis. (**E** and **F**) Relative protein of autophagy (LC3II and p62) were detected by Western blot and quantification analysis. (**G**–**J**) The expression of LC3II and p62 were further confirmed by immunohistochemistry and quantification analysis. The values are mean ± SD of three independent experiments. **P<0.01 vs NC group, ^##^*P*<0.01 vs IL-1β treatment group.

In order to investigate the effects of PAR2 on the production of inflammatory cytokines in chondrocytes induced with IL-1β, the supernatant of chondrocytes was collected. As described in Figure 4A,B, IL-1β could significantly elevate the production of TNF-α and Cox-2 compared with the normal group. However, PAR2 antagonist and Rapamycin could alleviate the production of TNF-α and Cox-2, and PAR2 agonist and 3-MA remarkably aggravated the production of TNF-α and Cox-2 when compared with the IL-1β group. Subsequently, Western blot analysis was performed to evaluate the protein expressions of MMPs in chondrocytes induced with IL-1β. As shown in Figure 4C,D, IL-1β could significantly up-regulate the protein expressions of MMP-1 and MMP-13 compared with the normal group. However, PAR2 antagonist and Rapamycin could down-regulate the protein expressions of MMP-1 and MMP-13, and PAR2 agonist and 3-MA remarkably promote the protein expressions of MMP-1 and MMP-13 when compared with the IL-1β group. Further, Western blot assays were carried out to determine the expressions of LC3-I, LC3-II and P62 related to autophagy in chondrocytes induced with IL-1β. As shown in Figure 4E,F, IL-1β could significantly increase the expression of LC3-I and decrease the expressions of LC3-II and P62 compared with the normal group. However, PAR2 antagonist and Rapamycin could significantly decrease the expression of LC3-I and increase the expressions of LC3-II and P62, and PAR2 agonist and 3-MA remarkably aggravated the expression of LC3-I and mitigated the expressions of LC3-II and P62 when compared with the IL-1β group. The results of immunofluorescence were consistent with that of western blot (Figure 4G-J). These data indicated that PAR2 could regulate the inflammatory response and the expressions of MMPs in chondrocytes induced with IL-1β which may be achieved by regulating autophagy of chondrocytes.

### Down-regulation of PAR2 inhibited the activation of p38 MAPK and NF-κB signaling pathway in chondrocytes induced with IL-1β

**Figure 5:**
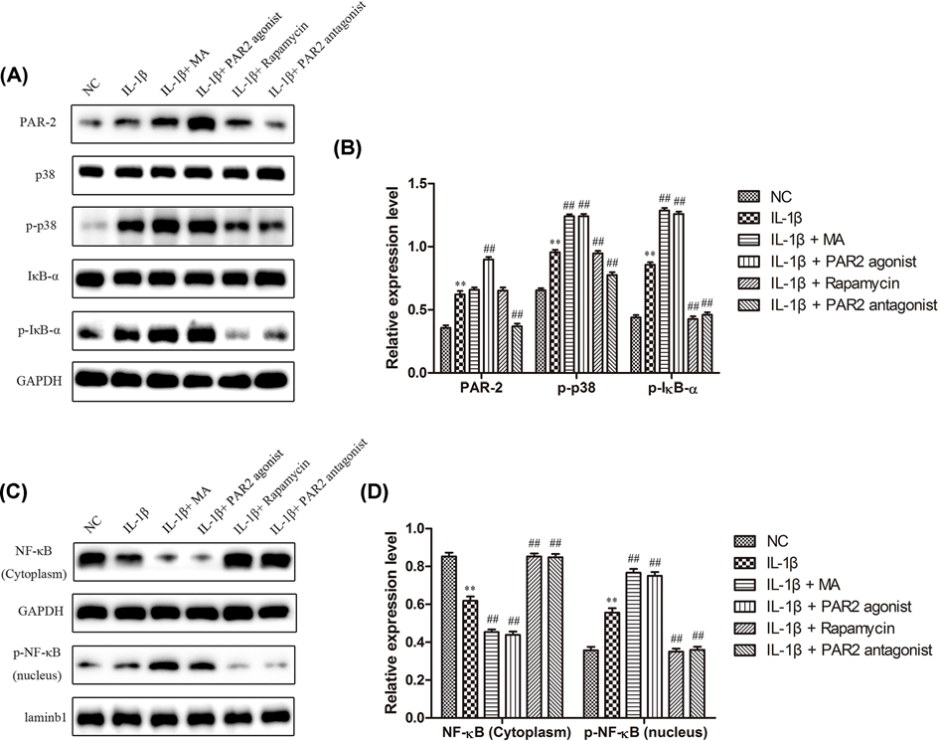
MAPK / NF-κB signal pathway participated in the effect of PAR2 on chondrocytes induced by IL-1β (**A** and **B**) The expression levels of PAR2, p38, p-p38, IκB-α, and p- IκB-α were determined by Western blot and quantification analysis. (**C** and **D**) The expression levels of p- NF-κB (nucleus) and NF-κB (cytoplasm) were determined by Western blot and quantification analysis. The values are mean ± SD of three independent experiments. **P<0.01 vs NC group, ^##^*P*<0.01 vs IL-1β treatment group.

P38 is the most important member of the MAPK family in controlling inflammatory response [32]. Both p38 MAPK and NF-κB signal transduction pathways are activated in articular cartilage and synovial cells of OA and high-expressed p38 can promote the expressions of NF-κB expression and further participate in synthesis and metabolism of cartilage matrix, playing an important role in the occurrence and development of OA [33]. In addition, the negative regulation of autophagy by p38 has positive effects on the condition of OA. Therefore, to explore whether PAR2 affected chondrocyte autophagy by regulating MAPK/NF-κB signaling pathway, Western blot assay was performed to examine the role of PAR2 in the expressions of MAPK/NF-κB signaling pathway. As shown in Figure 5A,B, IL-1β could significantly increase the expressions of PAR2, p-p38, p-IκBα and p-NF-κB (nucleus), and decrease the expressions of NF-κB (cytoplasm) compared with the normal group. However, PAR2 antagonist and Rapamycin could decrease the expression of expressions of p-p38, p-IκBα and p-NF-κB, and PAR2 agonist and 3-MA remarkably aggravated the expressions of p-p38, p-IκBα and p-NF-κB when compared with the IL-1β group, and the those effects were opposite about NF-κB (cytoplasm). These data indicated that PAR2 could affect the autophagy of chondrocytes maybe through MAPK/NF-κB signaling pathway.

### PAR2 affected proliferation in chondrocytes induced with IL-1β via regulation of MAPK/NF-κB signaling pathway

**Figure 6:**
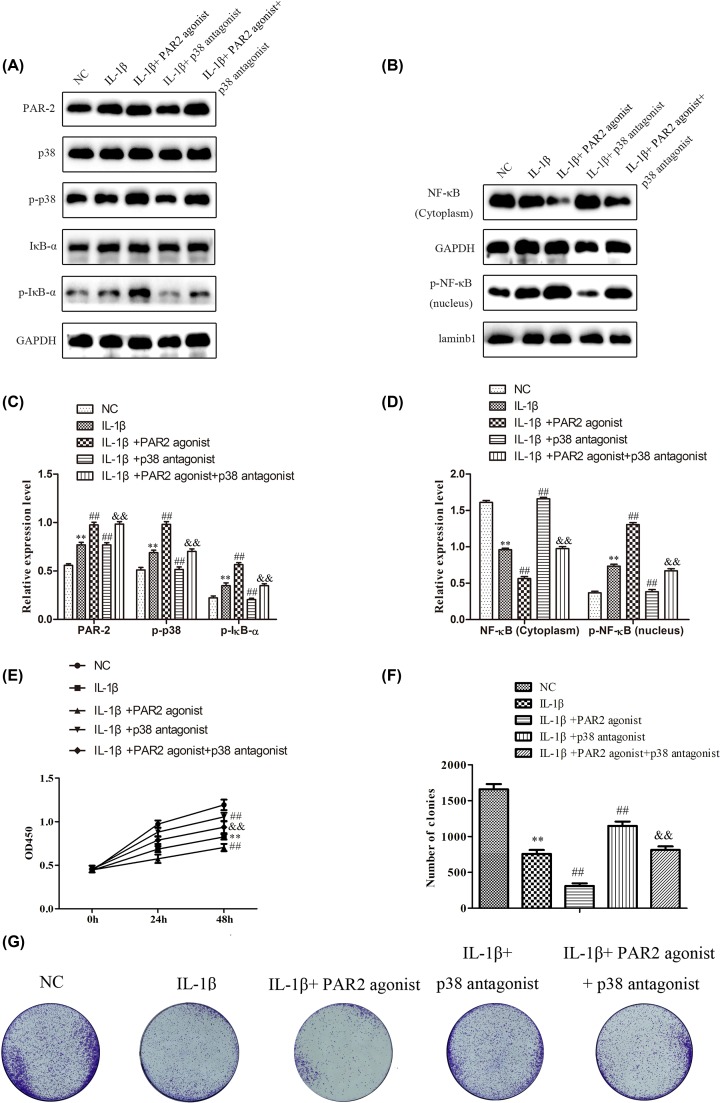
PAR2 regulated chondrocytes proliferation induced by IL-1β by MAPK / NF-κB signal pathway SB203580, a novel p38 MAPK inhibitor with selective activity against p38 MAPK, was employed to inhibit the p38 MAPK expression in chondrocytes induced by IL-1β. (**A**–**D**) The expression levels of PAR2, p38, p-p38, IκB-α, p- IκB-α, p- NF-κB (nucleus) and NF-κB (cytoplasm) were determined by Western blot and quantification analysis. (**E**) The cell viability was determined by CCK-8 assay. (**F** and **G**) The cell proliferation was determined by clony formation assay and quantification analysis. The values are mean ± SD of three independent experiments. **P<0.01 vs NC group, ^##^*P*<0.01 vs IL-1β treatment group, ^&&^*P*<0.01 vs IL-1β+ PAR2 agonist.

To further verify the involvement of MAPK/NF-κB signaling in proliferation of chondrocytes induced with IL-1β, SB203580, a novel p38 MAPK inhibitor with selective activity against p38 MAPK, was employed to inhibit the p38 MAPK expression, and Western blot was performed to detect the expression of MAPK/NF-κB signaling pathway. As shown in Figure 6A–D, the data showed that SB203580 could suppress the expression levels of p-p38, p-IκBα and p-NF-κB, and SB203580 could decrease the effects of PAR2 agonist on the expression of MAPK/NF-κB signaling. Then CCK-8 and colony formation assays were performed. The results of CCK-8 assay (Figure 6E) showed that SB203580 could significantly promote the proliferation of chondrocytes induced with IL-1β, and SB203580 could reverse the effects of PAR2 agonist on the proliferation of chondrocytes induced with IL-1β. Besides, as shown in Figure 6F,G, SB203580 could increase the colony formation of chondrocytes induced with IL-1β, and SB203580 could remarkably restore the effects of AR2 agonist on the colony formation of chondrocytes induced with IL-1β. These findings suggested that PAR2 could affect the proliferation and colony formation of chondrocytes induced with IL-1β may be through regulation of MAPK/NF-κB signaling pathway.

### PAR2 affected the production of inflammatory cytokines, the expressions of MMPs, and autophagy in chondrocytes induced with IL-1β via regulation of MAPK/NF-κB signaling pathway

**Figure 7:**
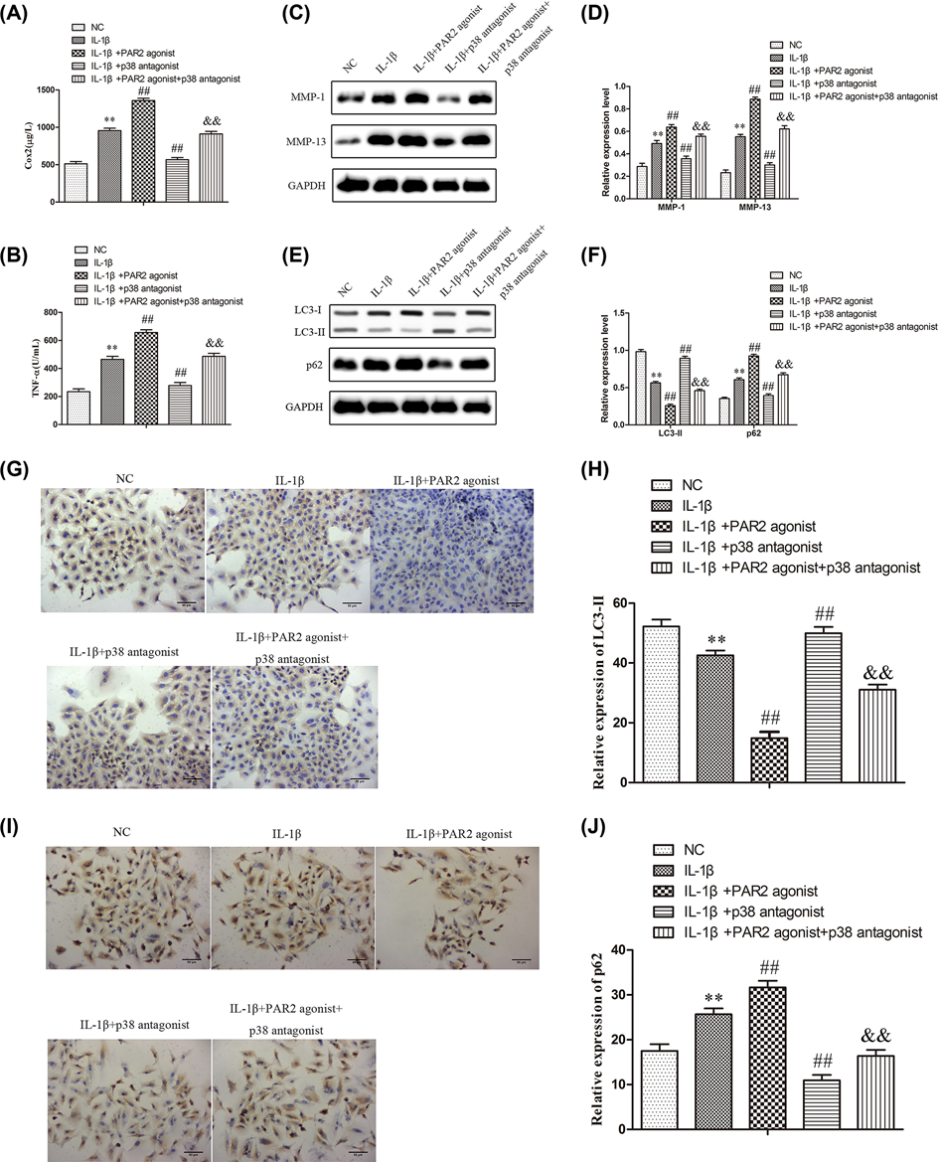
PAR2 regulates Inflammation factor, matrix protease, and relative protein of autophagy by MAPK / NF-κB in chondrocytes induced by IL-1β (**A** and **B**) Inflammation factor (Cox-2 and TNF-α) were determined using ELISA. (**C** and **D**) Matrix protease (MMP-1 and MMP-13) were detected by Western blot and quantification analysis. (**E** and **F**) Relative protein of autophagy (LC3II and p62) were detected by Western blot and quantification analysis. (**G**–**J**) The expression of LC3II and p62 were further confirmed by immunohistochemistry and quantification analysis. The values are mean ± SD of three independent experiments. **P<0.01 vs NC group, ^##^*P*<0.01 vs IL-1β treatment group, ^&&^*P*<0.01 vs IL-1β+ PAR2 agonist.

To further verify the involvement of MAPK/NF-κB signaling in the production of inflammatory cytokines, the expressions of MMPs, and autophagy in chondrocytes induced with IL-1β, SB203580 was employed to inhibit the p38 MAPK expression. Afterward, ELISA assay was carried out to evaluate the production of inflammatory cytokines, and the results of Figure 7A,B showed that TNF-α and Cox-2 levels were higher in chondrocytes treated with IL-1β than that in normal chondrocytes (NC) (*P* < 0.01), and TNF-α and Cox-2 levels were higher in chondrocytes treated with IL-1β and PAR2 agonist than that in chondrocytes induced with IL-1β (*P* < 0.01). Moreover, TNF-α and Cox-2 levels were lower in chondrocytes treated with IL-1β and SB203580 than than that in chondrocytes induced with IL-1β (*P* < 0.01), and SB203580 could reverse the effects of PAR2 agonist on the production of inflammatory cytokines in chondrocytes induced with IL-1β. In addition, Western blot assay was adapted to determine the expression levels of MMP-1 and MMP-13, and the results of Figure 7C,D indicated that SB203580 could significantly inhibit the expressions of MMP-1 and MMP-13 in chondrocytes induced with IL-1β and SB203580 could reverse the effects of PAR2 agonist on the expressions of MMP-1 and MMP-13 in chondrocytes induced with IL-1β. Further, Western blots were used to evaluate the expressions of LC3-II and P62 related to autophagy in chondrocytes induced with IL-1β. As shown in Figure 7E,F, the data showed that SB203580 could significantly increase the expression of LC3-II and inhibit the expression of P62 in chondrocytes induced with IL-1β and SB203580 could reverse the effects of PAR2 agonist on the expressions of LC3-II and P62 in chondrocytes induced with IL-1β. The results of immunofluorescence assays were consistent with that of Western blot (Fig 7G–J). These findings suggested that PAR2 could affect the production of inflammatory cytokines, the expressions of MMPs, and autophagy in chondrocytes induced with IL-1β may be through regulation of MAPK/NF-κB signaling pathway.

## Discussion

Osteoarthritis (OA) is a disease characterized by narrowing of joint space, osteophyte formation and cartilage degeneration, and is the most prevalent joint disease related to ageing [4]. Under the background of increasing biological aging, with the increasing incidence of OA, more and more attention has been paid to its harmfulness, which can lead to the loss of joint function and permanent disability, and it is the most important factor causing knee and hip weight-bearing joint dysfunction [34]. The pathogenesis of OA is very complex, but it is not fully understood. It may be related to many factors, including biomechanics, heredity, age and inflammation [4]. Therefore, a deeper understanding of the mechanism of the occurrence and development of OA is of great significance to guide the effective diagnosis and treatment of OA, and is also a medical problem that needs to be tackled urgently at home and abroad.

Advanced OA can involve the entire joint tissue such as synovium, cartilage and subchondral bone, among that, the destruction of cartilage tissues is an important process in the pathogenesis of OA, and is the main feature of pathological changes of OA [35,36]. Further, unbalanced metabolism and synthesis of cartilage tissues can lead to cartilage wear, matrix loss and chondrocyte death in the course of OA [37]. In the present study, we found that PAR2 antagonist could significantly improve the pathological changes of articular cartilage, decrease the production of IL-1β, TNF-α and Cox-2, and inhibit the protein expressions of MMP-1 and MMP-13 when compared with the OA group, while PAR2 agonist exhibited the opposite effects, which indicated that PAR2 may play an important role in the development and progress of OA, and down-regulation of PAR2 could significantly ameliorated OA.

Because there is only one kind of chondrocyte in cartilage, the study of chondrocyte is of great significance. Compared with normal chondrocytes, the anabolic ability of chondrocytes in OA is decreased, and the secretion of matrix factors, aging, oxidative stress and apoptosis are increased [38]. These characteristics promote the gradual aging and death of chondrocytes. Moreover, because the synthesis of cartilage matrix depends entirely on its only host cell, chondrocytes, the dysfunction of chondrocyte function and survival will directly lead to cartilage damage [39]. In articular cartilage, autophagy plays an important role in maintaining the balance and function of chondrocytes due to the low proliferation rate of chondrocytes. It has been found that the occurrence of OA is related to the decline of chondrocyte autophagy level [23]. Besides, animal studies have also found that inhibition of autophagy of chondrocytes significantly aggravated the degeneration of chondrocytes and cartilage in osteoarthritis and promoted the occurrence of OA [40]. Besides, some studies have found that in the early stage of OA, the expression of LC3 and atg5 in osteoarthritis chondrocytes were increased, suggesting that autophagy was enhanced [41]. However, in the late stage of OA, the expression levels of LC3 and atg5 in chondrocytes were decreased, the level of autophagy in chondrocytes was decreased, apoptosis was increased, and cartilage degeneration became serious, accompanied by the decrease of extracellular matrix synthesis, which indicated that the weakening of autophagy promoted the progress of OA [42]. Therefore, the present study was designed to explore the effects of PAR2 on the autophagy of chondrocytes. In OA rat model, we found that PAR2 antagonist could suppress the expressions of p62 and promote the expression of LC3-II when compared with the OA group, while PAR2 agonist exhibited the opposite effects, which indicated that PAR2 could regulate autophagy of chondrocytes in articular cartilage. Further, *in vitro*, our results showed that PAR2 antagonist could promote the proliferation and increase colony formation of chondrocytes induced with IL-1β, could alleviate the production of IL-1β, TNF-α and Cox-2, could obviously down-regulate the protein expressions of MMP-1 and MMP-13, and could decrease the expression of P62 and increase the expressions of LC3-II of chondrocytes induced with IL-1β. However, PAR2 agonist exhibited the opposite effects. The effects of PAR2 antagonist and PAR2 agonist on the autophagy of chondrocytes were similar to the effects of Rapamycin and 3-MA. The data indicated that PAR2 could affect the proliferation, inflammatory response and the expressions of MMPs in chondrocytes induced with IL-1β which may be achieved by regulating autophagy of chondrocytes.

Mitogen-activated protein kinase (MAPK) belongs to serine/threonine protein kinase family, and MAPK signal transduction pathway is one of the important pathways in eukaryotic signal transduction network, which can transduce extracellular stimulus signals into cells and their nuclei to further cause cellular biological effects. MAPK signaling pathway plays a key role in gene expression regulation and cytoplasmic function. Many studies have shown that MAPK signaling can participate in cell proliferation, differentiation, apoptosis and autophagy [43,44]. P38 MAPK as the most important member of the MAPK family in controlling inflammatory response has certain functions in influencing the differentiation of osteoblasts [25]. Autophagy induced by Rapamycin, an inhibitor of mTOR, was achieved by eliminating ROS, a mitochondrial by-product, to inhibit the production of IL-1β and IL-18 and the activation of caspase-1 [45]. A recent study has confirmed that PDK1 was highly expressed in chondrocytes of OA patients, and after knocking out PDK1, the expression of p38 was down-regulated in SW1353 cells and apoptotic cells, which was related to the decrease of IL-1β induced [46]. Moreover, a rabbit OA model study found that impaired autophagy was associated with early progress of OA, and negative regulation of autophagy by p38 played a major role in promoting OA disease [47]. Further, the high expression of P38 may promote the expression of NF-κB and participate in the synthesis and metabolism of cartilage matrix, which played an important role in the occurrence and development of OA [48]. The present study showed that PAR2 antagonist could decrease the expression of expressions of p-p38, p-IκBα and p-NF-κB, while PAR2 agonist exhibited the opposite effects. Moreover, to further verify role of MAPK/NF-κB signaling in occurrence and development of OA, we chose a p38 MAPK inhibitor, SB20358, to inhibit the MAPK/NF-κB signaling pathway, and we found that SB20358 could remarkably promote the proliferation and increase colony formation of chondrocytes induced with IL-1β, could alleviate the production of IL-1β, TNF-α and Cox-2, could down-regulate the protein expressions of MMP-1 and MMP-13, and could decrease the expression of p62 and increase the expressions of LC3-II of chondrocytes induced with IL-1β. Importantly, SB203580 could reverse the effects of PAR2 agonist on the functions of chondrocytes induced with IL-1β. These findings suggested that PAR2 could affect the production of inflammatory cytokines, the expressions of MMPs, and autophagy in chondrocytes induced with IL-1β maybe through regulation of MAPK/NF-κB signaling pathway.

**Figure 8:**
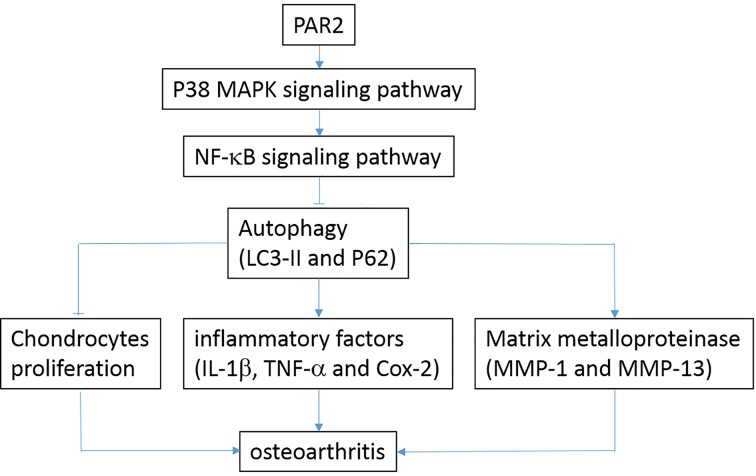
The relationship was described among PAR2 and OA PAR2 could activate MAPK signaling pathway, and then activate NF-κB signaling pathway, which cause the inhibition of chondrocytes proliferation, increase of inflammation factor (IL-1β, Cox-2 and TNF-α), and increase of matrix protease (MMP-1 and MMP-13). Those promote the progression of OA.

The current research is a preliminary study on about the effects of PAR2 on OA, and the other effects and underlying mechanisms of PAR2 in OA are not very clear. Based on the current findings, we will further explore the role of PAR2 in development and progress of OA both *in vivo* and *in vitro*, and explore the effective mechanisms of PAR2 in future. As summarized in Figure 8, the present data suggest that PAR2 could promote OA through decreasing autophagy via regulation of MAPK/NF-κB signaling pathway *in vivo* and *in vitro*. Taken together, PAR2 antagonist may be considered as a potential candidate to treat OA.

## Abbreviations

3-MA: 3-methyladenin
LC3: light chain 3
MMP: matrix metallopeptidase
OA: osteoarthritis
PAR2: protease-activated receptor 2
ROS: reactive oxygen species
ULK1: unc-51 like autophagy activating kinase 1
